# Dissected saccular aneurysm of the ascending aorta after percutaneous coronary intervention: A case report

**DOI:** 10.1016/j.amsu.2021.102178

**Published:** 2021-02-18

**Authors:** Ibrahim Hannoun, Albaraa Bara

**Affiliations:** Faculty of Medicine, Damascus University, Damascus, Syrian Arab Republic

**Keywords:** Case report, Dissected saccular aneurysm, Aneurysm, Aorta, PCI

## Abstract

**Introduction:**

and importance: Dissected saccular aneurysm of the ascending aorta is an extremely rare clinical entity. It has a greater risk for rupture due to its different histological structure compared to other forms of aneurysms; hence, it mandates urgent management.

**Case presentation:**

A 64-year-old retired pilot was referred to our hospital complaining of vague chest pain along with numbness in his upper limbs, Chest X-ray showed widening of the upper mediastinum, and transesophageal echocardiography showed a dilated ascending aorta measuring 7.5 cm.

**Clinical discussion:**

Transesophageal echocardiography and CT angiography confirmed the presence of a dissected saccular aneurysm in the ascending aorta, along with a dissection of the aorta that spanned the area between the dissected saccular aneurysm and the root of the innominate artery. As a surgical management, the ascending aorta was replaced with Dacron graft, the postoperative period was uneventful.

**Conclusion:**

We report this case to highlight that surgical repair should be offered immediately when a dissected saccular aneurysm is diagnosed.

## Highlights

• Dissected saccular aneurysm (DSA) is an extremely rare, life-threatening condition.
• DSA is thought to originate on top of focal lesions in the aortic wall.
• Hypertension and atherosclerotic disease are the main risk factors for DSA.
• PCI and Rapid Pressure Changes may be potential Cofactors for DSA.
• Elective surgical intervention should be considered in patients with DSA.

## 1 Introduction

True aneurysms could be divided into fusiform aneurysm, and saccular aneurysm. The more common fusiform aneurysm is defined as a bulging out on all sides of the artery, while saccular aneurysm is a ballooning out only on one side of the blood vessel [1].

Dissected saccular aneurysm is an extremely rare entity that resembles dissection with saccular aneurysmal dilatation, which is thought to originate on top of focal lesions in the aortic wall [2].

Because of its unique structure and shape, it is thought to have a greater tendency of rupturing compared to other forms of aneurysms; therefore, surgical intervention is usually advocated even if the aneurysm is asymptomatic [3].

Here we report a case of dissected saccular aneurysm of the ascending aorta in an ex-pilot who had undergone percutaneous coronary intervention (PCI) 15 years ago. This work has been reported in line with the SCARE criteria [4].

## 2 Case presentation

**Fig. 1:**
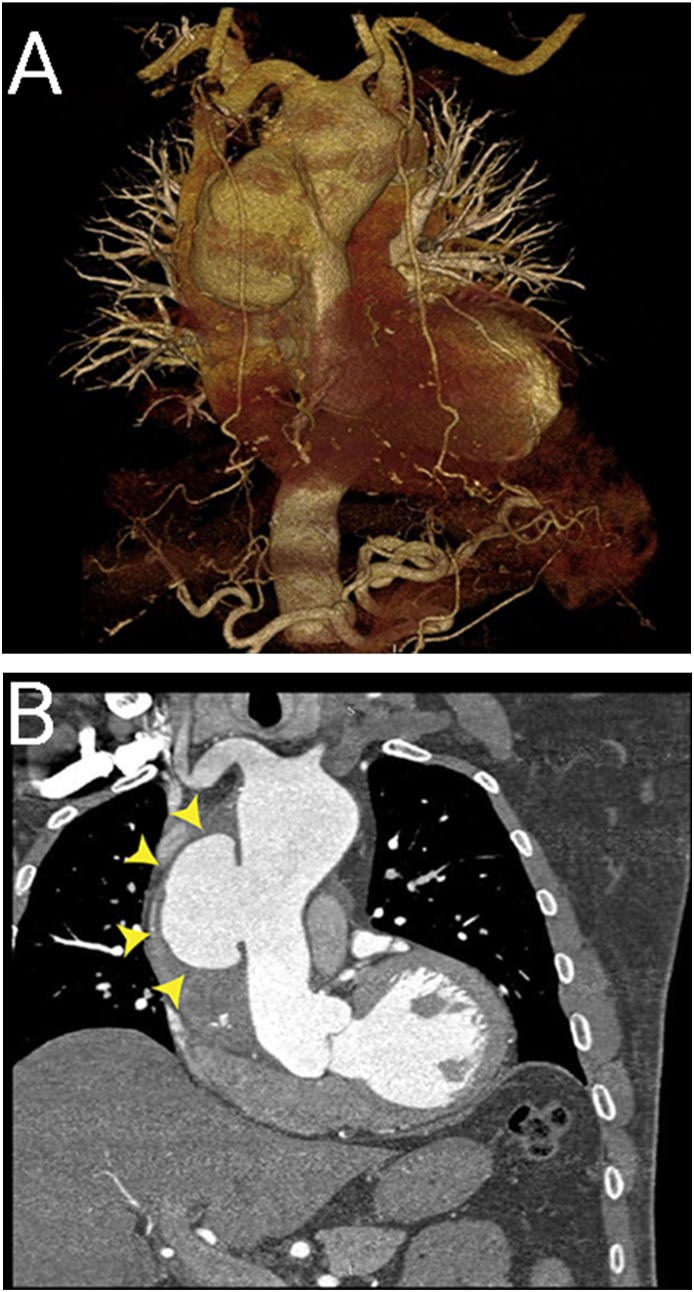
A. CT Angiography and B. Contrast enhanced CT scan showing a large dissected saccular aneurysm (yellow arrowheads). (For interpretation of the references to colour in this figure legend, the reader is referred to the Web version of this article.)

A 64-year-old retired pilot was referred from the community setting to our cardiac surgery center with complaints of vague chest pain in the precordial area that radiates to his left arm along with numbness in his upper limbs, the pain was accompanied by breathlessness and palpitations, the patient was non-smoker and had a history of hypertension diagnosed 20 years ago and previous coronary artery disease that was managed by PCI for both the Left Anterior Descending Artery (LAD) and the Left Circumflex Artery (LCX) 15 years ago. Physical examination was unremarkable and Chest X-ray showed widening of the upper mediastinum, and transesophageal echocardiography showed a dilated ascending aorta measuring 7.5 cm along with trace aortic valve incompetence as well as mild mitral valve incompetence, and a left ventricular Ejection Fraction of 56%. CT angiography of the chest confirmed the presence of a dissected saccular aneurysm measuring (64 × 41 mm) with its neck measuring 40 mm, along with a dissection of the aorta that spanned the area between the dissected saccular aneurysm and the root of the innominate artery (Fig. 1). Coronary angiography showed a sclerotic Right Coronary Artery (RCA) with a moderate constriction in its first third and a complete blockage in its second, along with a patent LAD and LCX stents.

The patient underwent surgery, by an experienced team with prior surgical experience with similar cases, under hypothermic circulatory arrest conditions. the aorta per se was sclerotic and the dissected saccular aneurysm was noted 5.7 cm distal to the aortic valve, with shelf-like protrusions at the lesion's proximal and distal edges, the lesion also contained some thrombi at its proximal aspect. Preserving the aortic valve, the entire diseased portion of the aorta was replaced with a 26 mm Dacron graft, and a 30 mm venous graft was prepared and implanted on the RCA. The postoperative period was uneventful; the patient is being followed up by transesophageal echocardiography as well as CT scanning the aorta every 3 months for the first post-surgical year and every year thereafter.

## 3 Discussion

Dissected saccular aneurysm is a rare clinical entity in comparison with other forms of aneurysms, with the dissected saccular aneurysms of the ascending aorta being extremely rare. This form of aneurysms is thought to have an increased risk of rupture and therefore should be managed when diagnosed regardless of its size [5]. Dissected saccular aneurysms are thought to form on top of a weakened area in the aortic wall which appear as a focal fibrosis that extends throughout the aortic intima and the middle third of the media, these lesions arise from previous ulceration of the intima [2]. Hypertension and atherosclerotic disease are the main risk factors for developing saccular aneurysms, other factors include trauma, infections including Mediastinitis, Syphilis and Tuberculosis, previous aortic surgery, Behçet's disease, and Takayasu arteritis [5,6].

To the best of our knowledge, only few cases of dissected saccular aneurysms of the ascending aorta have been reported [2]. we are reporting a case of a huge dissected saccular aneurysm in ex-pilot that has two major risk factors being hypertension and atherosclerotic disease, and had previously undergone PCI. We would like to draw attention towards the patient's previous PCI as being a potential cofactor in the pathogenesis of the lesion, as the catheter may cause microscopic damage to the aortic lining. The patient's occupation may also be a cofactor in the pathogenesis as pilots are subjects to rapid pressure changes due to the nature of their occupation.

## 4 Conclusion

Our case highlights the satisfactory outcome of electively repairing the dissected saccular aneurysms of the ascending aorta as well as drawing attention towards PCI and the piloting occupation as being potential risk factors contributing to the formation of said aneurysms.

## 5 Informed consent

Written informed consent was obtained from the patient for publication of this case report and accompanying images. A copy of the written consent is available for review by the Editor-in-Chief of this journal on request.

## Provenance and peer review

Not commissioned, externally peer-reviewed.

## Ethical approval

None.

## Sources of funding

None.

## Author contribution

AB: reviewed the literature, wrote the abstract, and the introduction.

IH: reviewed the literature, wrote the case presentation, and the discussion.

## Trial registry number

None.

## Guarantor

Mr. Albaraa Bara.

## Consent

Written informed consent was obtained from the patient for publication of this case report and accompanying images. A copy of the written consent is available for review by the Editor-in-Chief of this journal on request.

## Declaration of competing interest

All of the authors declared that they have no conflict of interest.
